# Hospital and Institutional News

**Published:** 1919-10-25

**Authors:** 


### HOSPITAL AND INSTITUTIONAL NEWS.

### GLASGOW'S UNIQUE OPPORTUNITY.

By the last train which left Euston and got straight through to Glasgow on the day when the railway strike first became effective as a train-stopper, we started for Glasgow and arrived without mishap. There we were strike-bound for nearly a fortnight. We were treated to samples of all kinds of Glasgow weather, from its worst to its best. We devoted our time to the study of the city, some of its great institutions, and searched for an adequate and attractive plan for the celebration of The Peace after the Great War, our wish being that any schemes, if adopted, should be fruitful in results for good to the city and its inhabitants. One whole day was spent at the new Royal Infirmary, which, after ten years' working experience and wear and tear, could claim, with justice, to be a great hospital capable of being made one of the most interesting of modern hospitals in the world. To secure this, without avoidable delay, it must be brought up to date and completed energetically. The appearance, hygienic atmosphere, and many attractive features the new Royal Infirmary buildings present, and the state in which they were found to be, gave cheer and satisfaction to the visitor. Indeed, the expert may make bold to affirm that, the New Royal Infirmary has before it a splendid future, providing its Managers take forthwith the steps which are urgently demanded by hygienic considerations and the Claims of An Ever-Increasing Nursing Staff.

### THE SECOND CITY IN THE EMPIRE?

These steps include the demolition of the superintendent's house and other old buildings, including portions converted into cubicles, which, though good of their kind, are out of date and must be scrapped. The vacated site will provide room for a New Nurses' Home of Ample Proportions, equipment and up-to-date plan. To clear the site of the old buildings, formerly the Lister wards, retaining The Operation Theatre and Lister's Top Ward, which the University of Glasgow will take under its care and erect on a site already in hand; the erection of that portion of the Admission Block which will provide for noisy and emergency cases, a most important and urgently needed section, required in justice to the patients in other parts of the hospital. These steps will accord with the high standard which led the Royal Infirmary Managers originally, to complete and efficiently equip a great infirmary which would accommodate 660 patients, and prove, when completed, a great attraction to all interested in the Perfecting of Hospital Buildings on a plan calculated to attract attention and bring honour to Glasgow from The Whole Hospital World. Once these steps are taken and completed Glasgow's Hospital Features will properly attract The World's Experts, whilst the city itself may become the Mecca of the World of Science, members of which will make their pilgrimage to the Lister Memorial Theatre and Wards, and so keep green the name of The Splendid Citizen of Glasgow, *whose system has saved more lives and prolongation of life* than probably any other man the world has known. We propose to deal with these questions fully in our next issue. We hope that this foreword may awaken A Real Interest in the *City of Glasgow* and secure that Here Celebration of the Peace on New Year's Day, 1920, will be Memorable for Ever in The Hostory of Great Cities.

### A GRAVE ALLEGATION.

The recent quarterly meeting of the Hospital Saturday Fund was memorable from the questions which were asked concerning certain hospitals, alleged to have refused treatment to the wives and children of strikers during the railway dispute. Sir Vezey Strong, who presided, said that the allegations had at once been the subject of inquiry. If they were true, the judgment of the Hospital Saturday Fund upon the hospitals which refused treatment could unhesitatingly be foretold. In his opinion any hospital authority who should try to distinguish between patients in equal need for any extraneous reason is a man of "most unfortunate" judgment. The Saturday Fund now as ever is doing its best to preserve efficiency and impartiality in those hospitals to which it contributes. We are informed that the questions referred to relate to one comparatively small special hospital only, and have no relation to certain or any other hospitals. In any case, the Executive is engaged at the moment in ascertaining the exact truth, and will no doubt report further when the result is known.

### THE CURRENT INCOME TAX YEAR.

The last day for sending in income-tax returns, on which abatements, exemptions, and reductions of rate can be legally claimed, is September 30. The income-tax officials are now in most parts of the country sending out their assessments, based upon the returns submitted to them; and it is necessary to point out that some of the statements in our article upon this subject (The Hospital, October 4, 1919, p. 11) are no longer wholly accurate, owing to changes made in the 1919 Budget since the article in question was written. Thus the allowance (tax free) for a wife is now £50, instead of £25, provided the total income is under £800. The abatement on income of £400 is now £120; between £400 and £600 the abatement is £100; and not exceeding £700 it is £70. The allowance in respect of children is now £40 for the first and £25 for each subsequent child; and this allowance can be claimed for children over 16 if they are in whole-time attendance at certain kinds of recognised higher education. If the income is between £800

and £1,000, children's allowance can still be claimed for two less than the actual number of children who would have been eligible for relief if the income were less than £800. There are also new provisions about dependent relatives and housekeepers. There are one or two other small changes in matters of detail: but in general the foregoing comprises the more important points of difference; and those now receiving their assessment papers will be well advised to see that they are obtaining the reliefs they are legally entitled to.

### A CHILDREN'S HOSPITAL.

A striking appeal is launched in the daily papers by the Hospital for Sick Children for the sum of one million pounds for the provision and endowment of a Children's Hospital Cottage City near London. The Great Ormond Street institution fairly claims to be the mother of children's hospitals, and by right of precedence and tradition the institution that should have the honour of founding a hospital city in the country for sick children which, it is stated, has for years been advocated by physicians and surgeons. The appeal is excellently drawn up and loses no point which will strengthen its attractiveness. That it is issued at a time when income tax is six shillings in the pound, and, on the authority of the Premier, not likely to be reduced, is not the fault of its promoters, for to wait for better times would be to wait for that which no one can see coming in the immediate future. The draftsman of the appeal does not overlook the parlous times we are passing through, for it is the comparatively prosperous, well, and happy who are addressed. Such will not grudge the children their city, their 500 fully endowed beds, the equipment and apparatus which is to be the last word in medical science, the daily motor ambulance, the educational facilities, the gardens and playgrounds, and the hostel for visiting parents. If they will also realise how precious child life especially is at the present moment, and if enough of them are reached the appeal that Lord Aberdare and his committee have so boldly issued will not fall upon deaf ears.

### TWO INTERESTING DINNERS.

The Prince of Wales has consented to preside at a festival dinner on behalf of the Great Northern Central Hospital. The date is not definitely fixed, but it is expected to be early in December. Another dinner of special interest is that arranged at Anderton's Hotel, Fleet Street, for Saturday, November 22, when a testimonial will be presented to Mr. A. W. Davis, the late secretary of the Hospital Saturday Fund. Applications for tickets should be made to his successor, Mr. Philip Inman, who, by the way, has received the congratulations of his friends upon his recent marriage to Miss May Dew.

### A RECENT ELECTION.

We have received information that the leading urologists of the allied and neutral nations have held a conference in the Faculté de Médécine, Paris, and elected Mr. E. Henry Fenwick, C.B.E., the well-known consulting surgeon to the London Hospital, President of the Société Internationale d'Urologie, in succession to Professor Guyon. This choice of a British surgeon is pleasing evidence of international amity in scientific circles, and we are glad to record our appreciation of the compliment implied.

### NATIONAL RAT WEEK.

The first of the National Rat Weeks opened on October 20, and if the efforts recently made to arouse active interest in this vermin problem bear fruit, there should soon be millions of these pests less in the country. The present arrangement should go far towards reducing the disease and destruction menace, but it is understood that drastic legislation is about to be brought to bear. Recently a Bill was introduced in the House of Lords but withdrawn in favour of a considerably stronger Government measure that will, it is hoped, pass into law during next session. Responsibility will then rest with the persons who harbour the vermin and who, if they do not efficiently deal with their own local problem of clearing premises, will find the matter taken up by the local authorities but at the offender's expense.

### THE ORIGIN OF INFLUENZA.

The recent address by Dr. Flexner in which he strongly suggests that the endemic focus of influenza is somewhere on the eastern border of Russia, and holds the view that medical science now possesses the resources to clear up a region which by its inaccessibility and its neglect has every twenty-five or thirty years originated waves of disease spreading over the world, has not been received without criticism. We read in a contemporary evidence that in 1889 the great influenza pandemic is reported to have started on the borders of European and Asiatic Russia, but we share the view very generally held that in regard to the recent outbreaks no such positive generalisation as Dr. Flexner's can as yet be made. There is, however, great point in the essential suggestion that disease of this type is best attacked at its endemic source, at a time indeed when no epidemic is occurring. The instance of yellow fever is typical of success in this direction. It needs but momentary reflection to recall a number of diseases where we do know the locality of origin and, although the work involved in the practical solution of such a problem would, in the majority of instances, be vast, it is well to bear in mind the possibility of thus ridding the world of a scourge, if coincident circumstances or a governmental opportunity should occur allowing of such undertakings at a not impossible expense.

### NEW CONSULTATIVE COUNCILS.

A draft of an Order in Council establishing Consultative Councils under the Scottish Board of Health Act will be laid before Parliament this session. In terms of the Act, before the Orderr can be made and come into operation, the draft of the Order must lie before each House for 30 days on which the House is sitting. The Order has been in draft for some time past, but during the Parliamentary recess the necessary procedure for bringing it into operation could not be carried through. It is proposed, under the Order, to set up four Consultative Councils in Scotland to advise the Board on the following matters respectively: (1) medical and allied services, (2) national health insurance (approved societies' work), (3) local health administration and general health questions, and (4) Highlands and islands. The members of the councils are to be appointed by the Board and are to be persons having practical experience of the matters for the purposes of which the particular council is established, and due regard is to be had to any special interests (including those of local authorities and of labour) which may be involved. The membership of any council is not to exceed twenty, and each council will include persons of both sexes. Provision is made for the councils appointing committees for special purposes and for the inclusion in such committees of persons who are not members of the council. The Board may convene joint meetings of any of the councils or committees of the councils. The councils are to meet at such times as they may, subject to the approval of the Board, determine, but it is specially provided that each council must meet at least three times in each year. The functions of the councils are set forth in the draft Order and include, *inter alia*, the consideration of and reporting upon questions which may from time to time be referred to them by the Board, including questions arising on draft Orders in Council, regulations, orders, etc., and questions involving consideration of any important principle and scientific difficulty affecting or incidental to the health of the people. Subject to the provisions of the Order each council has power to regulate its own procedure.

### A QUESTION FOR IERNE.

Except in the case of London's largest hospitals, the senior members of whose staffs have usually had wide experience elsewhere before returning to their old training-school, it is not the rule to appoint one of the sisters to be matron. A case can be made for and against these home-made appointments; but the plan has little to recommend it in the smaller hospitals, which need fresh blood and new brooms to keep them out of a rut, and perhaps more serious declensions if "an old gang" is left too long undisturbed. We notice, however, that an Irish hospital recently appointed the sister-in-charge to be matron, on the resignation of her former chief. Is this the custom in Irish hospitals? We rely on our correspondent Ierne for information, and would be glad to hear from the same quarter how far the plan succeeds in Ireland.

### WELSH BOARD OF HEALTH.

In accordance with the expectations held out to Welsh members during the discussions on the Ministry of Health Bill, arrangements have now been made to extend considerably the functions of the Welsh Board of Health. The work to be immediately transferred includes all the functions of the Housing Department of the Ministry of Health in Wales. Mr. A. Lloyd Thomas, the Housing Commissioner for Wales and Monmouth, becomes a member of the Welsh Board of Health, and his staff is transferred from the London to the Cardiff establishment. Arrangements have also been made to transfer to Cardiff an important part of the work of the Ministry in relation to public health. In view of the extent and importance of the functions involved the actual transfer cannot take place immediately, but the necessary preliminary arrangements have already been made.

### A CURIOUS LETTER.

The following extraordinary letter has been received by the superintendent of a well-known hospital, and we are much indebted to him for enabling us to reproduce it as it stands:

> "And I am ove 70 And I wish to sell my Busnes (business) as can I have put in the pape (paper) for sale as I can cure Bad legs cancer tumer and groths (growths) womb Absess gartia (goitre) I have cured 5 cases blood poisng (poisoning) and heart deceas (disease)
> Yours truly,
> Hirblist (Herbalist)."

The letter is addressed to the —— Cancer Infirmary.

The superintendent rightly describes this to be "a beautiful specimen of an English letter." We commend it to Mr. E. V. Lucas for his next selection of letters to be called, no doubt, "the last post." Before we laugh at the author, we should be scientific enough to inquire what his treatment is; and his apparent poverty must be his excuse for desiring to sell rather than to communicate his knowledge.

### THE DEAN OF HEREFORD.

The retirement of the Dean of Hereford from the Ward of the Herefordshire General Hospital, after twenty-five years of association with the management, is a loss that will be felt. He won a reputation for regular attendance, and the success of the local Hospital Sunday fund owed much to his care. In fact, it was he, we believe, who promoted the Hospital Sunday service.

### CANADIAN MILLIONAIRE AND BOOTLE HOSPITAL.

According to detailed reports from Canada, Bootle Borough Hospital is to receive a legacy of £100,000 under the will of the late Mr. Montgomery Cox, the Ottawa millionaire lumberman, who died three or four months ago. The hospital authorities have not yet received any official notification that the institution is amongst the beneficiaries and the first intimation reaching them has been through the public Press. Mr. Montgomery Cox was related to the family of Messrs. Robert Cox and Company, timber merchants, of Bootle. His total estate was reported to amount to close on $1,800,000. Of this sum about $400,000 is absorbed by various bequests, legacies, and annuities. The residue, it is reported, is to be divided in equal parts between the Bootle Hospital and St. Luke's Hospital, Ottawa. The Borough Hospital at Bootle has 103 beds and the ordinary annual expenditure, according to the most recent return, was £5,375. Allowing for heavy legacy duties, this bequest, should the news prove authentic, will put the hospital well upon its legs.

### AN EXAMPLE FROM CARLISLE.

Last Sunday, October 19, a service of thanksgiving was held in Carlisle Cathedral for doctors, nurses, V.A.D.s, members of St. John Ambulance, and other bodies who have helped in what may be conveniently called Red Cross work during the war. Official permission was given by the headquarters of the Red Cross for the wearing of uniform at the service, and the Dean and Chapter reserved seats in the stalls for medical men, and seats in the body of the choir for nurses. The occasion was very appropriate for such a service because October 19 was the Sunday following St. Luke's Day, when many doctors maintain the old tradition of attendance at special services in St. Paul's or local churches. The Rev. R. Symes, who was an Army chaplain at the front, preached the sermon. We should like to have been there, because doctors and nurses have few opportunities for corporate acts of ceremony, especially of this solemn character; and the whole history of the Middle Ages shows how valuable to the self-respect of professions, crafts, and trades are the ritual observances which their members make in common on special occasions. If that tradition is perishing, the loss is greater than men's indifference knows; and it becomes a duty to show the value of these observances whenever people exist who are enlightened enough to give an opportunity for them. The Dean and Chapter of Carlisle Cathedral deserve our respect accordingly.

### SOME NEW SECRETARIAL APPOINTMENTS.

Following upon the appointment of the new matron at the Kent and Canterbury Hospital, Canterbury, the committee have proceeded to fill the post made vacant by the resignation of the secretary, Mr. A. J. Lancaster, who is taking up work outside the hospital world. The choice of the committee has fallen upon Mr. F. P. Carroll, a gentleman who is well known in hospital circles. Mr. Carroll was for many years chief clerk at Charing Cross Hospital, and before taking a commission in the Royal Air Force, he held the post of secretary to All Saints' Hospital, Vauxhall Bridge Road. The new senior officers at Canterbury Hospital will have the advantage of working for an institution with an excellent record, and we are sure that the careful choice of the governors will ensure that the useful work of the hospital will continue to be carried out in the time-honoured efficient manner so well appreciated by the supporters of the institution. We learn that Mr. E. K. Pantling, late house clerk of the Great Northern Central Hospital has been appointed assistant secretary of Manor House Hospital, Golders Green. He is succeeded at the Great Northern by Mr. C. A. Johnson of the Royal National Hospital for Consumption, Ventnor.

### MOTOR SCOOTERS AND MEDICAL MEN.

The motor scooter has recently made its appearance on the streets and already a number of machines of various types and prices are being placed on the market by various firms. Thus, the *Skootamota* of Messrs. Campling, Ltd., 1 Albemarle Street, W. 1 is fitted with 1¼ h.p. engine, price £45; the *Autoglider*, with 2¾ h.p. two-stroke engine is catalogued at £40 by Messrs. Townsend, of Charles Street, Birmingham; the *Norlow*, designed by Sir Henry Norman and Dr. Low at £28 is manufactured by the Norlow Engineering Co., 38 Conduit Street, W. 1.; and the *Kingsbury*, with 2 h.p. engine, price £39, is obtainable from the London and Midland Motors, Ltd., 445 Oxford Street. Whether such machines will ever become a popular means of transit for medical men, it is difficult to say, but when the initial outlay, size, lightness, easy starting and low cost of running are considered, there is certainly a possible future in this direction —in fact, already one doctor in suburban London (at Walthamstow) is to be seen " scooting " to visit his patients.

### MESSRS. EVANS, SONS, LESCHER & WEBB.

On another page our readers will find a prospectus of the issue by this well-known firm of wholesale and export druggists of 150,000 new Ordinary shares of £1 each at a premium of 10s. each. In making a special feature of their publicity campaign of putting this issue before institutional and medical investors, Messrs. Evans, Lescher and Webb are acting wisely in their generation, for the reputation of their firm, which extends back to the reign of George III., is naturally less known to the ordinary public at large. For details of the issue and of the position of the company, our readers are referred to the prospectus itself; but it is perhaps as well to draw particular attention to the fact that the dividends are paid free of income tax, and are therefore more valuable than they appear at first sight.

### THIS WEEK'S DRUG MARKET.

The improvement in business continues. There is a good all-round inquiry and a fair amount of actual orders. The bringing of drugs into the schedules under the Profiteering Act is to be noted; it remains to be seen whether in the event of action being taken against retailers for overcharging, the cost of drugs will be traced to their origin—or at any rate to first hands on this side. In the meantime prices on the whole are well maintained, and in some cases the advancing tendency continues. Values of menthol, camphor, peppermint oil, for instance, are firmly maintained. Eucalyptus oil is in good demand at steady prices. Mercurials are unaltered, but in view of the unsettled state of the quicksilver market, calomel, corrosive sublimate, and other salts of mercury should be brought with caution. Quinine is still under control.

